# Challenges and Opportunities in Preclinical Research of Synthetic Cannabinoids for Pain Therapy

**DOI:** 10.3390/medicina56010024

**Published:** 2020-01-09

**Authors:** Bogdan Ionel Tamba, Gabriela Dumitrita Stanciu, Cristina Mariana Urîtu, Elena Rezus, Raluca Stefanescu, Cosmin Teodor Mihai, Andrei Luca, Gabriela Rusu-Zota, Maria-Magdalena Leon-Constantin, Elena Cojocaru, Bogdan Gafton, Teodora Alexa-Stratulat

**Affiliations:** 1 Center for Advanced Research and Development in Experimental Medicine (CEMEX), “Grigore T. Popa” University of Medicine and Pharmacy, 16 Universitatii Street, 700115 Iasi, Romania; bogdan.tamba@umfiasi.ro (B.I.T.); raluca.stefanescu@umfiasi.ro (R.S.); mihai.cosmin.teo@gmail.com (C.T.M.); andrei.g.luca@umfiasi.ro (A.L.); teodora.alexa-stratulat@umfiasi.ro (T.A.-S.); 2 Department of Rheumatology and Physiotherapy, “Grigore T. Popa” University of Medicine and Pharmacy, 16 Universității Street, 700115 Iasi, Romania; 3 Department of Pneumology, “Grigore T. Popa” University of Medicine and Pharmacy, 16 Universității Street, 700115 Iasi, Romania; 4 Pharmacology, Clinical Pharmacology and Algesiology Department, “Grigore T. Popa” University of Medicine and Pharmacy, 16 University Street, 700115 Iasi, Romania; rusu.i.gabriela@umfiasi.ro; 5 Medical Semiology Department, “Grigore T. Popa” University of Medicine and Pharmacy, 16 University Street, 700115 Iasi, Romania; leon_mariamagdalena@yahoo.com; 6 Morpho-Functional Sciences Department, “Grigore T. Popa” University of Medicine and Pharmacy, 16 University Street, 700115 Iasi, Romania; ellacojocaru@yahoo.com; 7 Medical Oncology-Radiotherapy Department, “Grigore T. Popa” University of Medicine and Pharmacy, 16 University Street, 700115 Iasi, Romania; gaftonbogdan@yahoo.com

**Keywords:** synthetic cannabinoids, analgesia, animal models, delivery systems, pain therapy

## Abstract

Cannabis has been used in pain management since 2900 BC. In the 20th century, synthetic cannabinoids began to emerge, thus opening the way for improved efficacy. The search for new forms of synthetic cannabinoids continues and, as such, the aim of this review is to provide a comprehensive tool for the research and development of this promising class of drugs. Methods for the in vitro assessment of cytotoxic, mutagenic or developmental effects are presented, followed by the main in vivo pain models used in cannabis research and the results yielded by different types of administration (systemic versus intrathecal versus inhalation). Animal models designed for assessing side-effects and long-term uses are also discussed. In the second part of this review, pharmacokinetic and pharmacodynamic studies of synthetic cannabinoid biodistribution, together with liquid chromatography–mass spectrometric identification of synthetic cannabinoids in biological fluids from rodents to humans are presented. Last, but not least, different strategies for improving the solubility and physicochemical stability of synthetic cannabinoids and their potential impact on pain management are discussed. In conclusion, synthetic cannabinoids are one of the most promising classes of drugs in pain medicine, and preclinical research should focus on identifying new and improved alternatives for a better clinical and preclinical outcome.

## 1. Introduction

Cannabis is among the oldest medicinal plants, often referred to in ancient books; it was used as a tincture or tea to alleviate rheumatic pain, intestinal constipation, disorders of the female reproductive system and even symptoms of malaria. Additionally, in India, it was recommended as an analgesic, anticonvulsant, hypnotic, tranquilizer, antispasmodic, diuretic, aphrodisiac and expectorant [1]. Its uses were more than just medicinal, since cannabis was considered an excellent fibre and oil source, and its fruits were used as food. A possible explanation for the versatility of cannabis is the incredible morphologic variability of the plant that is associated with varying concentrations of different bioactive compounds [2]. Collectively referred to as phytocannabinoids, there are over 100 lipid-soluble molecules that can be found in the resin produced by female cannabis plants, the best-known of which are tetrahydrocannabinol (THC) and cannabidiol (CBD). Following their discovery, isolation and purification, cannabinoids have become a subject of intense research due to their psychoactive properties and their increasingly widespread use as recreational drugs [3], which led to the first steps in unveiling the cannabinoid receptors. The endocannabinoid system is now believed to play an essential role in several physiological and pathological processes, such as learning, memory, cognition, motor control, anxiety, appetite, sleep, lipogenesis, insulin formation, fertility, vasodilation, muscle fibre formation, gastro-intestinal motility, nociception, immune modulation, bronchodilation and cancer [4].

Synthetic cannabinoids (SCs) were originally designed as probes of the endogenous cannabinoid system. Between 1970 and 1980, cyclohexylphenols and dibenzoypyrans were among the first types of SCs used in preclinical studies to localize cannabinoid receptors. However, published data were quickly used by illegal laboratories to create recreational drugs with a cannabis-like effect. As such, SCs have been extensively sold under various brand names (“Spice”, “K2”, “Cloud 9” or “Mojo”) and have consequently been perceived as extremely dangerous and toxic [5]. Since their discovery, it has become apparent that they are more potent than the natural compounds, thus partly explaining their toxicity. This is most likely due to their activity as full agonists and their higher affinity for cannabinoid receptors [6]. Indeed, acute, severe or unpredictable side effects have been reported following SC abuse, and hospital admission rates are consistently higher for SC use than for natural cannabinoids consumption. However, some of these effects are caused by improper manufacturing, uncontrolled packaging together with different, sometimes toxic excipients that are used in SC production. As such, the true potential for using SCs for medical purposes has been overlooked. Currently, only synthetic THC has entered clinical practice, being approved for treating chemotherapy-induced emesis and for increasing appetite in AIDS-related wasting disease. Additionally, one other cannabinoid (plant extract) has been recently approved for the treatment of seizures, secondary to two rare forms of epilepsy [7]. Nabiximols, a mouth spray made of a 1:1 mix of Δ-9-THC and the CBD extract from cloned chemovars, was licensed in the UK in 2010 and has also been approved in other European countries and Canada for the treatment of spasticity, neuropathic pain and bladder dysfunction [8]. Dronabinol, a synthetic THC compound with oral administration, was successfully tested both as an analgesic [9] and as a co-analgesic with opioids [10], amitriptyline, gabapentin or tramadol [11] in clinical studies. Similarly, ajulemic acid, a synthetic nonpsychoactive cannabinoid has shown anti-inflammatory effects in preclinical and clinical experiments and is undergoing phase III testing by means of a large clinical trial [12]. However, none of the newer, more potent SCs are currently approved as an analgesic, despite mounting evidence that links the endocannabinoid system to pain transmission and pain perception. Pain, defined as an unpleasant sensory and emotional experience associated with actual or potential tissue damage, or described in terms of such damage [13], remains a significant global burden, and current estimates report that worldwide one in five adults suffer from pain and one in ten is diagnosed each year with chronic pain [14]. Although there are several types of analgesic drugs available, long-term treatment is usually hindered by loss of efficacy and side-effects that impact the quality of life. Additionally, some types of chronic pain are notoriously difficult to treat. Neuropathic pain, a condition characterized by abnormal hypersensitivity to stimuli (hyperalgesia) and nociceptive responses to non-noxious stimuli (allodynia), affects 3–17% of the population and is not always alleviated by opioids [15]. Furthermore, the number of deaths secondary to opioid overdoses is on the rise, with more than 65% of drug-overdose deaths involving at least one opioid, most often morphine or fentanyl. Different interventions, such as improved drug monitoring programs and enhanced toxicology testing have not yielded successful results [16]. As such, a potential method to address the opioid epidemic could be the identification of new analgesic drugs that replace or reduce the need for chronic treatment with opioids.

SCs could represent a promising class of analgesics/co-analgesics for the treatment of chronic pain. Taking into account that numerous new variants of these drugs appear yearly, due to both legal and illegal research, there is a plethora of compounds to choose from when testing for analgesic effect. Worldwide, cannabis remains the most used illicit drug and expert projections estimate that its use will increase dramatically in the next 25 years [17]. A comprehensive review reported a steady increase in young cannabis consumers from 5% in 1967 to 64% in 1982 [1]. Similar trends are seen in Europe, with a 22.1% cannabis use prevalence in France and 177 new SCs identified and reported to the United Nations Office on Drugs and Crime in 2015 alone [18]. As such, understanding and emphasizing the potential medicinal uses of SCs and selecting those drugs that contain non-psychotropic cannabinoids for preclinical and clinical testing is of a paramount importance.

## 2. Pharmacological Mechanism of Action in Pain Transmission and Pain Perception

The endocannabinoid system is involved in numerous functions, acting as a broad-spectrum modulator for several pathways. It includes two major G-protein-coupled receptors—cannabinoid receptor 1 (CB1) and cannabinoid receptor 2 (CB2)—that can be found on different types of cells belonging to the nervous, cardio-vascular, hepatic, muscle-skeletal and reproductive systems. The endocannabinoid system has at least two known endogenous ligands, anandamide (AEA) and 2-arachidonoyl-glycerol (2-AG). Most SCs are able to act as agonists or antagonists on CB1 and/or CB2 receptors by bearing some structural similarity to AEA, 2-AG or different phytocannabinoids [19]. SCs are notoriously difficult to classify, because new compounds with various structural changes appear constantly, and because both controlled and illegal research contribute to the development of this class of drugs. Usually, those SCs created for legitimate scientific purposes have a serial designation, frequently related to the laboratory or the researcher responsible for their creation [20]. Some examples include the AM series (derived from chemical biologist Alexandros Makriyannis) and the JWH series (named for Dr. John W. Huffman) [21]. However, this does not offer any information regarding their structure or their effect on receptors and does not include the illicit drugs available on the street market [22]. One of the most-used classifications divides SCs according to structure as presented in Table 1 [21], where several SCs that will be discussed in this review are listed according to the structural class they belong to. Still, the classification has several limitations and some overlaps as well as being seriously hindered due to new classes and compounds that appear rapidly.

CB1 receptors are among the most widely expressed receptor proteins in the brain, and a particularly high concentration of CB1 receptors has been identified on presynaptic terminals [29]. Although almost ubiquitous in the nervous system, CB1 receptors are highly expressed in the hippocampus, basal ganglia, cerebellum, cortex, thalamus and periaqueductal grey matter [24]. The peripheral nervous system also has a high expression of CB1 receptors, especially in sympathetic nerve terminals, dorsal root ganglia and dermic nerve endings [30]. Activation of the CB1 receptor in the presynaptic terminal is associated with the inhibition of voltage-gated Calcium channels and the inhibition of the cAMP/PKA pathway, both of which are events that lead to a decrease in the release of neurotransmitters [4], thus decreasing pain transmission and pain perception. Additionally, CB1 receptors are also involved in synaptic plasticity [31] and can form homo- or hetero-dimers with several other classes of G-protein coupled receptors, such as an opioid or alpha-2-adrenergic [32]. CB2 receptors are mostly expressed on immune cells and are moderately expressed in several other peripheral tissues, such as the liver, adipose tissue, bone, cardio-vascular or reproductive system [4]. Although both CB1 and CB2 receptors are expressed in the central nervous system, only CB1 receptors are present in the peripheral nervous system [21] and are considered overall to be the ones responsible for altering neurotransmitter release and sensory perception.

In recent years, it has become more and more apparent that cannabinoids do not solely act on CB1 and CB2 receptors, but in fact modulate multiple pain targets [33]. In a similar manner, analogues of the natural compounds, acting as modulators on the endogenous cannabinoid system demonstrate possible therapeutic applications in many pathological conditions [34]. If natural products exert their medical benefits through the effect of Δ-9-THC, their analogues, however, show promising therapeutic use through the non-psychotropic cannabidiol [35] represented by its acid metabolite THC-COOH [36]. These observations led to the development of numerous SCs with individual pharmacological profiles and also specific receptor affinities [37], some of which have proven to be effective in pain management in the clinical setting (although, as previously stated, no SC is currently approved by FDA for pain management).

There is no current consensus regarding SCs’ effect on pain and nociception, because these different drugs have diverse structures and can act on enzymes, receptors and ion channels [38] or a combination of the three; additionally, they most likely exert at least part of their analgesic activity through modulating both postsynaptic neurons and presynaptic nerve endings, reducing neural inflammation [39].

The overlap between the opioid and cannabinoid receptor systems and the interactions between the two is believed to also be involved in the analgesic effect of cannabinoids [36]. Furthermore, the effect is also enhanced by the important anti-inflammatory role through the reduction of pro-inflammatory markers (TNF-α, iNOS and COX-2), increasing at the same time the anti-inflammatory effects of adenosine agonists by inhibiting A2A receptors [40], exerted most likely through CB2 receptor activation.

Moreover, activating PPARs, such as α and γ or TRPV1 ion channel, accounts not just for the anti-inflammatory and analgesic role, but also for their anti-tumorous and cardiovascular protective functions. Synthetic cannabinoids, such as ajulemic acid, CP55940, HU331, and JWH015, activate PPARγ, while others, such as WIN55,212-2, act over both PPARα and PPARγ [41].

Taken together, these multiple mechanisms of action at both peripheral and central sites in the nervous system represent various opportunities for research and development of analgesic SCs with minimal psychotropic effect. The best method for testing if this analgesic mechanism of action could indeed be translated to clinical practice is through well-designed, randomized placebo-controlled clinical studies. However, this is currently severely hindered due to ethical limitations [7] and prejudice regarding SC research. As such, using all preclinical tools available can significantly improve the chances of an effective synthetic cannabinoid that can be safely used in a clinical setting. Although the direct analgesic effect cannot be assessed in vitro, cell studies are useful for determining cell viability or genotoxicity, as well as for offering insight into hepatic drug metabolism. Animal studies represent a useful tool for short-term and long-term effects and offer insight into the drug’s analgesic potential, and subsequent pharmacokinetic and pharmacodynamic studies contribute to better characterise the new synthetic cannabinoid.

## 3. Main Methods for in Vitro Assessment of Newly Synthetized Cannabinoids

### 3.1. Cell Viability and Toxicity

The first screening test a drug must pass is cell viability, which is the proportion of live cells in a cell culture exposed to a new substance or compound. Similar tests are also used for the evaluation of cell proliferation [42]. Preliminary screening of different collections or batches of compounds for the assessment of the impact on cell proliferation or viability consists of the use of cell-based assays. Also, useful information such as signal transduction, receptor binding measurement and intracellular trafficking can be extracted through cell culture assays. There are many methods in use for cell viability estimation, and all of these use the number of viable cells as a quantification parameter, the best-known being the tetrazolium reduction (MTT) assay, the adenosine triphosphate (ATP) test and the COMET assay [43].

#### 3.1.1. MTT Assay

The MTT (3-(4,5-dimethylthiazol-2-yl)-2,5-diphenyltetrazolium bromide) tetrazolium reduction assay requires the incubation of MTT with the viable cells and those will convert the substrate (MTT) to a coloured product, quantifiable at a specific wavelength with the use of a plate reader; in most cases, there exists a direct proportion between live cells and colour intensity. The tetrazolium reduction assay was the first viability assay developed for a 96-well plate and suitable for high-throughput screening (HTS) [44]. Viable cells, metabolically active, will convert MTT into a purple-coloured formazan product with a maximum absorbance of around 570 nm, while the dead cells lose their ability to convert MTT into formazan (no colour is developed). Although the specific cellular mechanisms of MTT reduction are not completely understood, it is believed that either reaction with NADH or similar reducing molecules that transfer electrons to MTT are involved [45]. The assumption that MTT measures mitochondrial activity was mentioned in early papers and is still used [43]. Some parameters influence the signal generated in MTT assays and should take into account when to optimize the protocol: the number of viable cells and their metabolic status, concentration of MTT, and the length of incubation period. Although the MTT assay is often erroneously described as a test for proliferation evaluation, MTT reduction is a marker for cell viability and is dependent by intracellular metabolic status [46].

The amount of signal generated is dependent on several parameters, including: the concentration of MTT, the length of the incubation period, the number of viable cells, and their metabolic activity. All of these parameters should be taken into account when optimizing the assay conditions to generate a sufficient amount of product that can be detected above the background.

However, it is important to keep in mind that MTT reduction is a marker reflecting viable cell metabolism and not specifically cell proliferation. Tetrazolium reduction assays are often erroneously described as a way to measure cell proliferation without the use of proper controls to confirm the effects on metabolism [47]. Both SCs and phytocannabinoids have been tested by means of the MTT assay, most often when assessed as potential anti-cancer drugs [48]. A recent study assessed the potential cardiotoxicity of SCs by exposing myoblasts to different concentrations of JWH-210, JWH-030, JWH-250 and RCS4. The results indicate that all of the SCs, especially JWH-030, decreased cell viability as assessed by MTT, at doses as low as 0.1 μM, most likely act through CB2 receptors [49]. Similarly, the nephrotoxicity of JWH-122 and THJ-2201 was confirmed in vitro by means of the MTT assay, whereas the underlying mechanism, which seems to be mitochondria-related, was identified through ATP assays.

#### 3.1.2. ATP Assay

In HTS applications, the quantification of ATP by firefly luciferase is the most commonly applied method for cell viability estimation, being largely accepted as a robust marker for viable cells. In SC research, this can be a very useful tool due to the extremely large number of compounds that are currently available and can thus be screened in order to identify the least toxic SC for a specific type of cell. Additionally, the ATP assay is very useful for excluding cytotoxicity as the mechanism through which the drug exerts its effect. Studies showing the anti-proliferative effect of cannabinoids usually use a cell viability assay to show that the drug inhibits the formation of new cells instead of inducing cell death [50]. Other studies have used the ATP assay to demonstrate potential anti-cancer uses of these drugs in cervical and oral tumours [51,52].

The method is based on the fact that when membrane integrity of the cells is lost, their ability to synthetize ATP is also lost, and endogenous ATP-ases act on the cytoplasmic ATP and rapidly deplete it. Even though luciferase was used for decades for the purpose of ATP measurement, recent advances into assay design have improved the protocol through the use of a single reagent and the persistence of the luminescence for hours. The stable version of the luciferase turned out to be more resistant to luciferase inhibitors found in libraries of small molecules, thus allowing for the development of a robust HTS assay [53]. Cell viability evaluation by the ATP assay is the fastest, most sensitive and least prone to artefacts than other method. Additionally, the luminescent signal stabilizes 10 min after the addition of the reagent and becomes half quenched after 5 h [54]. Variations into ATP assay sensitivity are much more due to pipetting errors than a result of the chemistry behind the assay.

#### 3.1.3. Genotoxicity Evaluation of Synthetic Cannabinoids by the Comet Assay

More recently, potential damage of the genetic material of a new drug undergoing a preclinical assessment prior to clinical use has become more and more of a concern due to long-term side-effects that have been discovered in substances otherwise deemed safe by in vivo and in vitro tests. Single-cell gel electrophoresis (COMET assay) is a simple method for the quantification of DNA strand breaks in eukaryotic cells and consists of embedding cells into the agarose on a microscope slide, and lysing with a detergent and high salt, thus forming nucleoids with supercoiled loops of DNA linked to the nuclear matrix. Electrophoresis performed in alkaline conditions reveals structures, such as comets, observed by fluorescence microscopy, with the intensity of the comet tail relative to the head indicating the number of breaks in the DNA. Comet scoring is performed using the OpenComet plugin for ImageJ (CellProfiler software with a comet pipeline can also be used). The results are expressed as a % tail DNA, % head DNA, Tail Moment and Olive Tail Moment.

The ability to measure heterogeneity in response to DNA-damaging agents was first tested on cells exposed to the cancer chemotherapeutic drug, bleomycin [55]. A wide range of appearances in the comets showed that some nuclei contained large numbers of strand breaks, whereas others were undamaged. If the negatively charged DNA contained breaks, DNA supercoils were relaxed, and broken ends were able to migrate toward the anode during a brief electrophoresis. If the DNA did not have any damage, the lack of free ends and the large size of the fragments prevented migration. Determination of the relative amount of DNA that migrated provided a simple way to measure the number of DNA breaks in an individual cell [56], thus offering a measure of a drug’s genotoxicity. Both phytocannabinoids and SCs have been assessed in several studies by means of the COMET method, and the available data indicate that CBD and cannabidivarin can cause DNA damage and chromosomal aberrations in human hepatoma and buccal epithelial cells at concentrations similar to those seen in recreational users of cannabis [57]. The assessment of XLR-11 and RCS4, as well as frequently used SCs, yielded similar results for human lymphocytes and buccal- and lung-derived human cell lines, generating the hypothesis that long-term consumption of these SCs might lead to respiratory tract tumours [58]. Due to the large number of available SCs, it is possible to identify, by means of the COMET assay, a non-genotoxic compound that can be later used in in vivo tests to determine its analgesic efficacy.

### 3.2. In Vitro Assessment of Synthetic Cannabinoid Metabolism

Because there are a lot of different SCs on the market, and because the rate at which new SCs emerge is constantly growing, studying their metabolism is very important, both for identifying potential toxic metabolites and also for choosing the correct test for detecting illegal substance abuse.

In vitro models are useful for characterizing SC metabolites, and there are several currently in use. Among them, incubating the drug with human hepatocytes faithfully reproduces the in vivo process due to the availability of both phase I and phase II liver enzymes. Additionally, such tests can also determine the drug’s half-life (the time it takes the parent drug to disappear after being incubated with human hepatocytes). As an added benefit, the results can also be compared with urine samples from individuals that have used the SC illicitly, and through this, the concurrence rate between in vitro-assessed metabolites and metabolites eliminated by urinary excretion can be determined. Successful testing has been reported for different SCs, such as AB-PINACA/5F-AB-PINACA, AB-FUBINACAFDU-PB-22/FUB-PB-22 and NM-2201 [59]. Another option for evaluating SC metabolism is by incubating the drug with liver microsomes and endoplasmic reticulum vesicles that contain enzymes responsible for most of the drug clearance mechanisms. Although significantly easier to use and cheaper than human hepatocytes [60], the concordance rate between in vitro metabolites and urine-assessed by-products is, predictably, lower. Nonetheless, this is a very useful screening tool to identify powerful metabolites of a new SC and subsequently testing them in vivo.

## 4. Animal Models for Assessing the Analgesic Effect of Synthetic Cannabinoids

After successful in vitro testing, any new SC should undergo in vivo assessments that further confirm safety and offer a measure of its efficacy. A constant exploration of drug development relies intensely on animal models to assess the efficacy, safety profile and tolerability of agents toward possible targets [37]. Various behavioural tests have been validated for analgesia testing and substantial preclinical evidence is available in the literature demonstrating SCs’ effects against pain perception [39,61].

SCs have been tested on rodent models for syndromes of the cardiovascular and respiratory systems, cancer treatments or metabolic conditions. As analgesics, various behavioural experiments have provided substantial preclinical evidence of SC’s effectiveness in different acute pain models [39,61]. However, most of the research has been focused on chronic pain states, where SCs have yet again proven effective, especially for neuropathic and chronic inflammatory models (Table 2) [62,63,64,65,66,67,68,69,70,71,72].

Cannabinoids have a central, spinal or peripheral site of analgesic action. This provides precise delivery sites. Synthetic cannabinoid compounds that have similar targets, such as Δ-9-THC, induce analgesic and antinociceptive responses in animal models of acute, neuropathic and inflammatory pain through specific interaction of CB1 and CB2 receptors [62,64,73,76].

CB1 receptors are abundantly found in various brain regions [77]. In diverse preclinical models of pain states, direct administration of synthetic cannabinoids in these regions of the brain was demonstrated to have both antinociceptive and antihyperalgesic effects, probably by increasing descending inhibition [73,76]. The concept of spinal cord mediation of the analgesia is supported by the CB1 receptor’s location in the superficial dorsal horn level, an intimate area implicated in processing pain. Opioids are often administered through epidural or intrathecal ways and the laboratory data supports the effectiveness for similar routes of delivery for synthetic cannabinoids [39].

The most consistent evidence for a peripheral analgesic site of action is derived from local administration of doses of synthetic cannabinoids that are not active in systemic delivery. Via precise interaction of cannabinoid receptors, the peripheral administration of synthetic cannabinoids reduces the formalin pain response (a model of inflammatory pain). The mechanism does not appear to be completely elucidated but may be based on a reduced release of peripheral neuron neuropeptides or by modulating the primary afferent sensitization of other types of molecules [74].

Recent studies in rodent models of pain have also revealed a CB 2 receptor-associated analgesia, without side effects of central nervous system associated CB1 receptors. One hypothetical mechanism of action appears to be through CB2 receptor activation by inhibiting both degranulation of mast cells and migration of the neutrophils, which leads to a reduction of inflammation [72,74,76]. CB2 receptors are more prevalent, but not completely peripheral, and are located in cells with immune function [78]. Nevertheless, cannabinoid receptor type 2 may be distributed in the central nervous system in pathological conditions [79].

The main issues when it comes to creating a novel G protein-coupled receptor agonist for management of pain include biodistribution, physical dependence, and tolerance. In the case of CBD and THC, the gaps in understanding appear to be due to the fact that their pharmacokinetics (PK) is dependent on the route of administration [37]. Both absorption and bioavailability differ in inhalatory, oral ingestion or transdermal delivery, especially due to CYP450 metabolism in the first-pass hepatic metabolism and due to their lipophilic proprieties, regards of crossing the blood-brain barrier. Thus, inhaled CBD has a bioavailability of 2–56% and achieves peak plasma concentration in 5–10 min, as compared to oral administration, which reaches its highest concentration in up to six hours and has a bioavailability of under 20% [40]. In preclinical neuropathic pain models, even the administration of effective CB1 agonist doses leads quickly to tolerance. Moreover, physical dependence can be proven, in the same way, after repeated administration of low doses of the CB1 agonist. In contrast to CB1-mediated analgesia, the tolerance to CB2-associated analgesia does not appear to be induced, in treatments lasting about 7 days, and there are no signs of physical dependence [72]. This absence of the potential for tolerance towards the analgesic properties of a CB2 agonist raises the question as to whether CB2 agonist-associated immunomodulation will also be persistent, and if so, whether this will be unfavourable.

Nevertheless, it is essential to determine whether the effectiveness of this multi-targeted approach reported in in vivo models of chronic pain could be translated into humans and is not specific for the studied species. An important cautionary tale comes from available data from previous clinical studies comprising volunteers with experimentally induced pain or clinical trial cases, which suggest that cannabinoids are not truly effective in acute pain and may be useful only in chronic pain syndromes. The increasing failure rate of compounds in clinical testing, although they all have favourable data in preclinical research, may be attributed to potential factors such as: species variances in receptor sequences and signalling ways, differences between research methodology and results recorded in the studies or lack of selectivity of the ligands used in rodent models of the targeted validation. These discrepancies could explain why the translation of CB2 agonists have failed from preclinical experiments to human treatments [39]. In addition, the outcomes of a clinical pain study may be influenced by factors that include the type of pain, study design or target population [61,80].

The current findings highlight the fact that systemic administration of synthetic cannabinoids will not have a major role in management of pain, but they can find a niche role in some pain states, such as a neuropathic condition, where existing therapies are deficient [39,62,69].

## 5. Interaction of Synthetic Cannabinoids with Other Drugs

Since the interaction of drug molecules usually happens with multiple targets, and the existence of unintended drug–target or drug–drug interactions, especially in pathologies requiring multi-drug management, studying the interaction of known or in development synthetic cannabinoids with other drugs is paramount [81].

In preclinical studies, as well as in clinical practice, any new analgesic drug is first assessed as a single agent. If the drug is effective, different combinations most often with opioids, are tested in order to identify synergic effects but also potentially deleterious interactions. For cannabinoids, one study concluded that adding vaporized cannabis to opioid treatment does not influence the plasma concentration–time curves for morphine or oxycodone but improves pain management significantly (27% decrease) by comparison with opioid administration alone, underlining the possibility of opioid treatment at lower doses with fewer side effects [82]. Most often, in clinical studies assessing cannabinoids as pain medicine, enrolled patients were already receiving chronic analgesics, such as tramadol, amitriptyline, gabapentin or morphine. Adding drugs from the cannabinoid family usually had a synergic effect, and no cumulative toxicities were noted [8,10,11]. The medical research has been focused on interactions between the cannabinoid-opioid and “opioid-sparing effect” of cannabinoids. Since medicinal marijuana has a reduced potential for addiction, dependence and abuse, it can be considered as a possible substitute of narcotics. In relation to the findings above, other studies have taken into consideration the role of medicinal cannabis as a factor in decreasing opioid-induced mortality since it was demonstrated that the presence of medical cannabis as a treatment option leads to lower annual opioid overdose mortality rates [83].

In preclinical models, several types of cannabinoids have been shown to decrease neuropathic nociception; there are several on-going clinical studies that evaluate medicinal cannabis preparations for the treatment of neuropathic pain. For example, CR701, a synthetic cannabinoid receptor, is being tested for effects over chemotherapy-induced neuropathy, inflammation and pain [84]. Unfortunately, studies that assess potential interactions between cannabinoids and drugs used to treat other neuropathic-generating conditions, such as multiple sclerosis, diabetes, human immunodeficiency virus, or herpes zoster infection [85], are lacking.

## 6. Toxicity of SCs

The topic of SCs’ toxicity is quite controversial. As previously stated, due to their effect as full agonists, SC abuse can be very dangerous, especially since CB1 and CB2 receptors are located in several tissues and organs. While the side-effects following the use of natural cannabinoids have been assessed quite thoroughly [86], the fast pace at which new SCs are developing makes it difficult to completely characterize both short-term and long-term side effects for each SC in particular. Reports from units that treat cases of acute SC intoxication indicate a plethora of short-term effects, including, but not limited to, cardiac toxicity (including cardiac arrest) [87], gastro-intestinal changes/hyperemesis [88], acute rhabdomyolysis, malignant hyperthermia, stroke, and seizures [89]. Long-term side effects are less studied, although exposure to SCs or THC during adolescence has been associated with schizophrenia, and repeated use increases the incidence of cognitive impairment and other mental disorders in later life [90]. Non-psychotropic chronic side effects of SCs include an increased risk for myocardial infarction (due to an increased heart-rate) and a decrease in fertility [91]. However, because this class of drugs is usually engineered to have different affinities for different types of cannabinoid receptors, potential analgesic SCs need not have this side-effect profile. For example, SCs that limit the agonistic effects to the CB2 receptor and peripherally located cannabinoid receptors, offer the possibility to use them as drugs with anti-inflammatory and antinociceptive effects without psychotropic side effects and an improved safety profile especially since drug-related toxicity effects are observed at higher concentrations than those considered the therapeutic dose [84].

Since cannabinol (CBN) has a 90% decrease in psychoactive effects as compared to Δ-9-THC, whereas CBD has no such proprieties, the side effects of opiate withdrawal are not present [37], and most of the adverse effects associated with SC administration are limited to moderate symptoms (headache, fatigue, dry-mouth) and are transient [84,92]. Due to the paucity of CB1 receptors in the respiratory centre of the brainstem, cannabis exposure is not typically associated with respiratory depression [24]. Although treatment with cannabinoids has been associated with some side-effects, most often after oral administration, the majority of these effects are mild and short-lasting [39].

In animals, LD50 of oral THC is 800–1900 mg/kgc in rats, and other tested doses from 3000 mg/kgc up to 9000 mg/kgc in dogs and monkey led to no deaths. Also, no acute fatal cases were reported in humans, although THC may trigger myocardial infarction. While some SCs are available on prescription, and are up to 800 times more active than THC, most are still undergoing clinical trials for pain treatment or other pathologies [36].

Even if the short-term usage adverse effects of SCs are mild to moderate and well tolerated, data is still required regarding long-term usage of such drugs, especially on cognition, the immune system, fertility and pregnancy, since both in vivo and in vitro studies have demonstrated that cannabis consumption may disrupt the hypothalamus-pituitary-gonadal axis, spermatogenesis and sperm function [34]. However, studies of up to a two-year SC treatment reported only cannabinoid-related adverse events [39].

## 7. Liquid Chromatography–Electrospray Tandem Mass Spectrometry Methods for the Quantitation of Synthetic Cannabinoids

The synthetic cannabinoid naphtalen-1-yl-(1-pentylindol-3-yl) methanone (JWH-018) was selected as a criterion for scientific literature search because it is the first synthetic cannabinoid for which a validated quantitation method employing liquid chromatography–tandem mass spectrometry was reported. The first study performed by Jörg Teske and collaborators presented a method for extracting JWH-018 from human serum and the results obtained by analysing samples from two healthy volunteers who smoked the incense “Smoke”. The sample pre-treatment, extraction method and the chromatographic column used for the separation of the compounds extracted are presented in Table 3, together with the limit of detection and lower limit of quantitation obtained. Taking into consideration the lower limit of quantitation of 0.21 ng/mL, the serum sample collected from volunteer 1 at 5 min after smoking indicated a concentration of 8.1 ng/mL, and this value decreased to 0.41 ng/mL in the serum prepared from the blood collected after 3 h [93].

In the study performed by Sebastian Dresen and collaborators, a different extraction method followed by detection and quantitation was developed and validated (Table 3). The method was used for seven other synthetic cannabinoids, of which four (JWH-015, JWH-073, JWH-081, JWH-200) are classified in the naphtoylindoles group; one was classified in the phenylacetylindole group (JWH-250), and methanandamide is a derivative of the endogenous cannabinoid anandamide. The method was also employed for the detection of JWH-019, JWH-020 [94]. In the study carried out by Ammann and collaborators, JWH-018 and 24 other synthetic cannabinoids were analysed using the same sample pre-treatment and extraction procedure. For JWH-018 and 21 other synthetic cannabinoids the mass spectrometric analysis was performed in positive mode, while for 3 substances, the mass spectrometer was operated in negative mode [95].

In another study, JWH-018 and 29 other synthetic cannabinoids were analysed using an improved sample pre-treatment and extraction procedure (Table 3) in comparison with the method published in the year 2011 [94]. The method was applied to the analysis of 833 serum samples collected, in which 227 positive results were obtained. In 80% of the positive samples, the researchers identified JWH-210, and in 64% JWH-122 [96]. Another method developed and validated by Ambroziak K. and Adamowicz P. allows for the analysis of 72 synthetic cannabinoids, which are classified into different chemical groups (Table 3) [97]. Stefan Kneisel and collaborators reported the development and validation of JWH-018 and 27 other synthetic cannabinoids from the neat oral fluid method, which has the advantage of being non-invasive if compared with the analysis of samples prepared from blood [98]. Kacinko S. L. and collaborators reported a procedure for analysing JWH-018, JWH-019, JWH-073 and JWH-250 in human whole blood [99]. Poklis J.L. and collaborators developed a method for the analysis of blood collected from mice. The mice were exposed to smoke produced by burning an incense product, and blood samples were collected and analysed at 20 min and 24 h after exposure [100].

Limited data is available on the relationship between the use of specific laboratory techniques and the detection of synthetic cannabinoids in clinical settings. Nevertheless, general PK/PD applications are safe to be inferred. Liquid chromatography coupled to mass spectrometry (LCMS) is an essential pharmacological tool, due to its capability to provide information on the quantity of certain chemical compounds after extraction from complex biological matrices such as biological fluids. For some chemical compounds, the quantity detected by a mass spectrometer coupled to a high-performance liquid chromatograph can be as low as 50 femtograms. Due to its high sensitivity, low sample consumption, fast and simultaneous analysis of chemical compounds, LCMS represents an important technique for plasma levels detection and quantification (i.e., in pre-clinical and/or clinical research, in patient toxicology investigations, etc.) and for investigations of dose–effect relations for synthetic cannabinoids (SC). Future studies may further fine-tune more specific detection and measuring techniques for the SC and their influence on the clinical data.

## 8. Improving the Solubility and Physicochemical Stability of Synthetic Cannabinoids

Although available data indicate that phytocannabinoids and SCs could be useful in managing several types of painful conditions, their hydrophobic nature has made them difficult to work with. Different cannabinoids have different bioavailability, and it varies significantly depending on the mode of administration. If smoked or inhaled, the resulting exposure depends on several factors, such as depth of inhalation, puff duration and breath-hold; additionally, it also varies between heavy users and occasional smokers [34]. The oral ingestion of such molecules is very limited due to their poor solubility in the hydrophilic intestinal milieu. Furthermore, THC, CBD and several of their metabolites with oral use are susceptible to first pass mechanisms, with slow, irregular and unpredictable absorption that can be further influenced by gastric pH and food [101,102]. These absorption hindrances render the molecules extremely unpredictable in terms of bioavailability [103]. Because of their poor absorption and reduced bioavailability, oral administration of cannabinoids has an additional drawback, requiring several administrations a day, another inconvenience for patients with difficulty in swallowing.

The highly lipophilic behaviour of cannabinoids underlines the crucial role of formulation in achieving the expected therapeutic effect. Different strategies to improve the solubility and physicochemical stability of active principles have been experienced, with favourable results in terms of pain management. Another aspect to be considered regarding the formulation of synthetic cannabinoids is their reduced stability. Recent research conducted by Pacifici and collaborators showed that the stability in the aqueous solution was so short that extemporaneous tea preparation was recommended, whereas in case of oil preparation, a loss of around 20% from its initial concentration was detected for all the tested cannabinoids during the first 14 days of storage [104].

To overcome the limitations given by the structure and physical-chemical properties of the cannabinoid molecules, several formulations and delivery systems were designed, produced and tested with various results regarding drug bioavailability and efficacy. A selection of these systems, corresponding to the most recent research, will be presented below.

### 8.1. Inclusion of Cannabinoids in the Cavity of a Cyclodextrin

Cyclodextrins are cyclic carbohydrates derived from starch that contain 6, 7 or 8 glucopyranose units, which are referred to as α-, β- and γ-cyclodextrin, respectively. Each cyclodextrin subunit has secondary hydroxyl groups at the 2 and 3 positions and a primary hydroxyl group at the 6 position. Inclusion in a cyclodextrin is one of the most well-known formulations aimed at enhancing the aqueous solubility of hydrophobic compounds. The cyclodextrins can be imagined as truncated cones having hydrophilic outer surfaces, while the central cavities are lipophilic [105]. In aqueous solutions, these cavities may accommodate hydrophobic organic compounds or part of their structure. This results in the so-called inclusion complexes which are stabilized by hydrophobic interactions and does not encompass the formation of any covalent bonds [106].

Kingsley and collaborators patented an invention that provides aqueous formulations containing at least one cannabinoid, such as THC or CBD and a sulfoalkyl ether cyclodextrin (SAE-β-CD). The SAE-CDs are a class of negatively charged cyclodextrins, which vary with respect to the nature of the alkyl spacer, the salt form, the degree of substitution and the starting parent cyclodextrin. The anionic sulfobutyl ether substituent significantly enhances the aqueous solubility of the underivatized parent cyclodextrin. The sodium salt of sulfobutyl ether derivative of β-cyclodextrin, having on average 7 substituents per macrocyclic ring (SBE7-β-CD), is commercially available (CAPTISOL^®^ cyclodextrin).

The liquid formulations of sulfoalkyl ether cyclodextrin-containing cannabiniods are substantially clear, sterilizable, chemically and physically stable. These solutions do not precipitate upon dilution with distilled water or other pharmaceutically acceptable liquid carriers. Solutions may be formulated to be either dilutable or non-dilutable with water, at an ambient temperature or under specific conditions encountered in clinical practice [107].

The invention in question affords a commercially feasible product that can be manufactured and stored in aqueous media at a wide range of physiologically acceptable pH values and concentrations of cannabinoid without important precipitation of the active principle.

### 8.2. Transdermal Delivery of Cannabinoids

Research performed by Stinchcomb and collaborators provided transdermal delivery systems loaded with cannabinoids through an occlusive form (i.e., a patch) to relieve destructive, undesirable side effects and escape from the gastrointestinal tract, as the first-pass metabolism of the drug. The active principle should be at least one cannabinoid from the group consisting of cannabinol, cannabidiol, nabilone and levonantradol, (−)-HU-210, (+)-HU-210, 11-hydroxy-Δ9-THC, Δ8-THC-11-oic acid, CP 55,940, and R(+)-WIN 55,212-2. The formulation also consisted of at least one agent to increase permeability from the group consisting of diethylene glycol monoethyl ether, propylene glycol monolaurate, a caprylocaproyl macrogolglyceride, an oleoyl macrogolglyceride, and an oleyl alcohol. Moreover, this development affords an occlusive body able to deliver cannabinoids, containing the following elements: an impermeable backing, a microporous membrane of a controlled transport rate, a permeation enhancer through the cavity, a viscous flowable gel-type placed between the backing and the membrane inside the cavity, where the cannabinoid drug and the permeation enhancer are immobilized. To achieve an increased concentration of cannabinoids or cannabinoid metabolites in a subject, the contact is also important between the device and the patient’s skin [108].

### 8.3. Enhancing Trans-Corneal Penetration

Although the role of cannabinoids in glaucoma therapy is clearly understood, there is still a lack of commercial products on the pharmaceutical market. The most common neuroprotective prescriptions to cure optic neuropathies are formulated as eye drops, but in fact less than 5% of the active principle penetrates the cornea [109], and subsequently, the amount of the drug in the formulation should be much higher than required.

Kabiri and collaborators (2018) developed a nanoparticle-based product able to improve the transport of the bioactive compound across the cornea and to obtain a sustained-release system, by extending the time when the drug concentration reaches therapeutic levels [110]. This research consists of preparation of a stimulus-responsive hydrogel, loaded with nanoparticles, forming in situ, as a controlled delivery system of cannabinoids inside the aqueous humor of the eye. The hydrogel composition encompasses hyaluronic acid (HA) and methylcellulose (MC). The nanoparticles reported by this research are composed of poly (ethylene oxide) (PEO) and poly (lactic acid) (PLA), providing them with amphiphilic properties. The nanoparticles synthesized and described within this work were loaded with cannabigerolic acid (CBGA), known as a neuropathic pain reliever, with successful effects in various chronic optic neuropathies, including glaucoma [111,112]. The formulation was optimized to obtain a sol-gel transition around 32 °C, which is approximately the same as the surface of the eyeball. The product was evaluated both in a rheometer under conditions simulating the ophthalmic surface and in vivo on porcine eyeballs, showing an increase in transcorneal penetration greater than 300% against control formulation. Additionally, the nanoparticle loading hydrogel can also be formulated as a liquid, with multiple benefits, including facile dosing [110].

### 8.4. Advanced Pro-NanoLiposphere (PNL)

PNL pre-concentrate was developed as a lipid-based Self-Emulsifying Drug Delivery System to enhance the therapeutic efficacy of cannabidiol (CBD) and Δ-9-THC in various medical conditions. The PNL delivery system comprises triglycerides of medium chain, a co-solvent, surfactants and a natural absorption enhancer incorporated inside: piperine [103]. These molecules are alkaloids and phenolic compounds that inhibit certain phase I and phase II metabolic processes. Available data show that oral administration of CBD-piperine-PNL leads to a 6-fold increase in AUC when compared to a free CBD solution. Pharmacokinetic experiments of THC-piperine-PNL showed a similar behaviour with a 9.3-fold increase in the AUC when compared to the free THC solution [113].

### 8.5. An Oral Formulation of THC and CBD—PTL401

PTL401, based on a self-emulsifying drug delivery system, aimed at avoiding the “first pass” effect, was developed by Cherniakov and collaborators [114]. Accordingly, the PTL401 product yielded 1.6-fold higher maximum plasma concentration than the equivalent dose of a reference commercial product (Sativex^®^, oromucosal spray), for both cannabinoid active principles. Their relative bioavailability was also superior (131 and 116% for CBD and THC, respectively), while the Tmax values were considerably smaller using both CBD and THC (1.3 h on average for PTL401 vs. 3.5 h for the spray). This product is based on a PNL (lipid nanoparticle) formulation of the aforementioned cannabinoids. The isotropic mixture encloses THC and CBD at a ratio of 1:1, lipids, a small amount of surfactants and a co-solvent. The nanoparticles (~30 nm) comprise a lipid core, allowing for cannabinoid solubilization and also facilitating the enterocyte penetration. The lipid core of the PNL product is composed of medium chain triglycerides that empower a supplementary absorption route of cannabinoids by means of the lymphatic system. It was proved that the cannabinoids are transported across the intestinal mucosa by the chylomicrons and then undergo lymphatic absorption [115].

The PTL401 formulation can be loaded up to 100 mg per capsule and has shown good stability at room temperature. The preclinical studies also revealed that the PNL loaded with THC or CBD enhance the oral bioavailability of both molecules up to 6 times, due to increased absorption rather than a decreased elimination rate [109].

### 8.6. Liposomal and Micelle Formulations of Cannabinoids 

Liposomal and micelle formulations of Cannabinoids for oral administration have improved dissolution and enhanced bioavailability and absorption, without producing gastrointestinal irritation. The cannabinoids or cannabinoid analogues can be: natural, synthetic, semi-synthetic compounds, or mixtures thereof. The proposed aqueous micelle suspensions of cannabinoids reach a maximum concentration in active principles of 2 g/L. The stable aqueous micelle suspension comprises an amount of 0.25–2% (*w*/*v*) stabilizer, such as: guar gum, xanthan gum, cellulose, hyaluronic acid, polyvinyl pyrrolidone (PVP), alginate, chondroitin sulfate, poly gamma glutamic acid, gelatin, etc. [116].

An innovative method for obtaining stable aqueous micelle suspension of cannabinoids, devoid of phospholipids and cholesterol, comprises the steps of (a) dissolving the cannabinoids in ethanol; (b) injecting the ethanol cannabinoid solution into distilled water to obtain a micelle cannabinoid aqueous suspension; and (c) removing the ethanol from the cannabinoid aqueous suspension, thereby producing a stable aqueous micelle suspension loaded with cannabinoids, with a diameter size ranging between 50 and 1000 nm.

The same research group obtained a highly concentrated liposomal formulation, having a maximum concentration in cannabinoids of 50 g/L with the diameter size of liposomes ranging between 200 and 400 nm. In such formulations, the lipophilic membrane comprises a maximum of 50% phospholipids of the total composition, of which about 26% are phosphatidylcholine, about 10% phosphatidylethanolamine, about 13% phosphonophospholipids and about 1% of other phospholipids [116].

## 9. Conclusions

SCs represent an incredible new class of drugs, with a variety of psychotropic and non-psychotropic effects. The preclinical data indicate that they could be very effective in different types of chronic pain, including neuropathic and visceral pain, both of which are extremely hard to treat in clinical practice. Creating new SCs that have most of the benefits of this drug class without long-term neurological effects represents a challenge for all laboratories.

Because several industry players are interested in developing SCs, researchers have many structural variants to work with and can thus identify a stable and effective SC with analgesic properties. Although biomedical research on the application of cannabinoids in clinical practices produces about 1000 papers per year, only a small number of products are currently available on the pharmaceutical market, along with some others, which are in the stage of clinical or animal studies. High-quality preclinical research, where all in vitro and in vivo steps are followed accordingly, significantly increases the probability of the drug being effective in human studies, thus adding a new class of analgesics to our treatment repertoire. Additionally, the opioid-sparing effect of SCs could significantly decrease the number of deaths caused by opioid over-use and lead to optimal pain management with minimal side effects. The prejudice that currently surrounds SC research needs to be overcome in order to establish these drugs as innovative analgesics.

## Figures and Tables

**Table 1:** Classification of SCs according to structure [23,24,25,26,27,28].

SC Class	Representatives	SCSC Class	Representativess
Aminoalkylindoles	AM-1241 JWH-018 JWH-210 JWH-081	Naphthoylindoles	WIN55,212 JWH-015 JWH-019 JWH-020 JWH-073 JWH-122 JWH-200
Adamantoylindoles	AKB48	Phenylacetylindoles	JWH-250
Benzoylindoles	RCS-4	Tetramethylcyclopropyl ketone indoles	XLR-11
Cyclohexylphenols	CP-47497 CP-47497 C8 CP55940	Quinolinyl ester indoles	PB-22
Dibenzopyrans	HU-210	Indazole carboxamide compounds	AB-FUBINACA AB-PINACA
Naphthoylpyrroles	JWH-030		

SC: Synthetic cannabinoids.

**Table 2:** Synthetic cannabinoids in different animal models of pain.

Pain States	Author	Synthetic Cannabinoid	Route of Delivery	Animal Model	Results
**Neuropathic pain conditions**	Herzberg et al. [62]	R(+)-WIN 55,212-2 mesylate	systemic	a rat model of traumatic injury of the sciatic nerve	antinociceptive effects similar to those of THC
	Pascual et al. [63]	WIN 55,212-2	systemic	a rat model of a neuropathic condition induced by paclitaxel	sustained inhibition of the thermal hyperalgesia and allodynia determined by paclitaxel
	Liang et al. [64]	WIN 55,212-2	systemic	a rat model of trigeminal neuralgia	attenuate allodynia and hyperalgesia
	Yamamoto et al. [73]	JWH133	intrathecal	a mouse model of partial sciatic nerve ligation	decrease mechanical allodynia
	Kinsey et al. [66]	O-3223	systemic	different types of mice pain models	antinociceptive effects without the development of tolerance or apparent cannabinoid behavioural effects
**Inflammatory pain**	Hanus et al. [67]	HU-308	systemic	formalin murine model of pain	attenuates formalin-evoked pain behaviour
	Clayton et al. [68]	GW405833	local and systemic	carrageenan model of inflammatory pain	decrease carrageenan-evoked hyperalgesia and hind paw swelling
	Elmes et al. [69]	HU210 and JWH-133	systemic	carrageenan model of inflammatory pain in rats	attenuates inflammatory hypersensitivity and swelling
	Nackley et al. [70] and Quartilho et al. [71]	AM1241	local or systemic	carrageenan model of inflammatory pain	reduces paw oedema and attenuates the progression of carrageenan-induced hyperalgesia
**Cancer pain**	Deng et al. [72]; Li et al. [74]; Rahn et al. [75]	AM1710	systemic	a mouse/rat chemotherapy-induced neuropathy model	blocked chemotherapy-induced allodynia without generating tolerance, physical withdrawal and other side effects of the central nervous system’s associated CB1 receptors
	Rahn et al. [76]	WIN55,212-2 (R,S)-AM1241	intrathecal	a rat vincristine-induced neuropathy model	suppressed vincristine-evoked mechanical allodynia without causing catalepsy

**Table 3:** Studies reporting the quantitation of the synthetic cannabinoid JWH-018 by LC-ESI-MS/MS.

Compound for Which LC-ESI-MS/MS Method Was Searched in the Scientific Literature	Number of Additional Synthetic Cannabinoids Analyzed	Biological Fluid and Quantity	Sample Pre-Treatment	Synthetic Cannabinoid Extraction	Chromatographic Column Used for the Chromatographic Separation	Limit of Detection (LOD) and/or Lower Limit of Quantitation (LLOQ) for the Compound JWH-018
JWH-018 [93]	0	Human serum, 200 μL	100 μL water, 20 μL internal standard and 10 mg NaHCO3	1 mL Hexane/ethyl acetate 99+1 (*v*/*v*)	Luna C18 column	LOD 0.07 ng/mL and LLOQ 0.21 ng/mL
JWH-018 [94]	7	Human serum, 1 mL	20 μL internal standard, 0.5 mL borate buffer (pH 9)	1.5 mL of n-hexane/ethylacetate 90:10 (*v*/*v*)	Luna phenyl hexyl column	LLOQ 0.1 ng/mL
JWH-018 [95]	24	Human blood, 100 μL	10 μL internal standard, 0.2 mL trizma buffer	1 mL 1-chlorobutane containing 10% isopropanol	Eclipse XDB C18 column	LLOQ 0.5 ng/mL
JWH-018 [96]	29	Human serum, 1 mL	10 μL internal standard, 0.5 mL carbonate buffer (pH 10)	1.5 mL of n-hexane/ethyl acetate 99:1 (*v*/*v*)	Luna phenyl hexyl column	LOD 0.02 ng/mL, LLOQ 0.1 ng/mL
JWH-018 [97]	71	Human blood, 200 μL	20 μL internal standard	600 μL of ice-cold acetonitrile	Kinetex C18	LOD 0.02 ng/mL, LLOQ 0.1 ng/mL
JWH-018 [98]	27	Neat oral fluid, 200 μL	10 μL internal standard	600 μL of ice-cold acetonitrile	Luna phenyl hexyl column	LOD 0.02 ng/mL, LLOQ 0.2 ng/mL
JWH-018 [99]	3	Human blood, 200 μL	25 μL internal standard, 200 μL, saturated sodium bicarbonate, 200 μL saturated sodium chloride	3 mL 99% hexane/1% ethyl acetate	Acquity UPLC HSS T3 C18 column	LOD 0.006 ng/mL, LLOQ 0.1 ng/mL
JWH-018 [100]	1	Mouse blood, 250 μL	750 μL drug-free human blood, 50 μL internal standard	2 mL of ice-cold acetonitrile	Zorbax Eclipse XDB C18 column	LLOQ 1 ng/mL
